# 4-CPA (4-Chlorophenoxyacetic Acid) Induces the Formation and Development of Defective “Fenghou” (*Vitis vinifera × V. labrusca*) Grape Seeds

**DOI:** 10.3390/biom11040515

**Published:** 2021-03-30

**Authors:** Zhenhua Liu, Yan Wang, Wenjiang Pu, Haifeng Zhu, Jinjun Liang, Jiang Wu, Liang Hong, Pingyin Guan, Jianfang Hu

**Affiliations:** 1 College of Horticulture, China Agricultural University, Beijing 100193, China; liuzhenhua93@163.com (Z.L.); tracywang0104@163.com (Y.W.); puwenjiang_yuanlin@163.com (W.P.); zhf51@outlook.com (H.Z.); liangjinjun1989@163.com (J.L.); wj619432057@163.com (J.W.); hl1006436066@163.com (L.H.); 2 Molecular Cell Biology, Botanical Institute, Karlsruhe Institute of Technology, 76131 Karlsruhe, Germany; pyguan@cau.edu.cn

**Keywords:** grape, auxin, defective seeds, integument, seed coat

## Abstract

For some horticultural plants, auxins can not only induce normal fruit setting but also form fake seeds in the induced fruits. This phenomenon is relatively rare, and, so far, the underlying mechanism remains unclear. In this study, “Fenghou” (*Vitis vinifera* × *V. labrusca*) grapes were artificially emasculated before flowering and then sprayed with 4-CPA (4-chlorophenoxyacetic acid) to analyze its effect on seed formation. The results show that 4-CPA can induce normal fruit setting in “Fenghou” grapes. Although more seeds were detected in the fruits of the 4-CPA-treated grapevine, most seeds were immature. There was no significant difference in the seed shape; namely, both fruit seeds of the grapevines with and without 4-CPA treatment contained a hard seed coat. However, the immature seeds lacked embryo and endosperm tissue and could not germinate successfully; these were considered defective seeds. Tissue structure observation of defective seeds revealed that a lot of tissue redifferentiation occurred at the top of the ovule, which increased the number of cell layers of the outer integument; some even differentiated into new ovule primordia. The qRT-PCR results demonstrated that 4-CPA application regulated the expression of the genes *VvARF2* and *VvAP2*, which are associated with integument development in “Fenghou” grape ovules. Together, this study evokes the regulatory role of 4-CPA in the division and continuous redifferentiation of integument cells, which eventually develop into defective seeds with thick seed coats in grapes.

## 1. Introduction

One of the most obvious phenomena of parthenocarpy is the formation of completely seedless fruits; for instance, an auxin application can induce aborted seeds in tomatoes, cucumbers, and pears [1,2]. Auxin was the first plant hormone to be discovered, and it has important regulatory effects on plant cell division and differentiation, lateral root formation, tropism, flowering, and embryonic development [3,4,5]. In particular, auxin accompanies another plant hormone, gibberellin (GA; both synthesized and accumulated in the fertilized ovaries after pollination/fertilization), both of which modulate the plant fruit set [6,7]. The current model suggests that GA biosynthesis is under the control of auxin in the early stages of fruit development; for instance, exogenous auxin application induces GA biosynthesis, whereas GA treatment does not activate the auxin response in Arabidopsis and tomato [8,9]. Furthermore, the exogenous application of those hormones on ovaries or genetic mutations in genes involving hormone synthesis (such as overexpression of genes of auxin biosynthesis or silencing of the gene *DELLA,* which have been considered master negative regulators of GA signaling) can induce pollination-independent fruit set [6,10,11]. GA treatment can stimulate embryo or fertilized egg abortion in grapes and some citrus varieties [12,13]. However, in the past, our laboratory discovered that a seed-like product is formed under the action of the plant growth regulator 4-CPA in grapes [14]. Although the underlying mechanism of this phenomenon remains elusive, we speculate that it might link to a special signaling pathway of 4-CPA application and seed formation in grapes.

In past decades, auxin has been noticed and identified as a molecular trigger of seed development (e.g., embryo identity and structure, embryo sac development, and gametophyte cell differentiation and specification). However, the mode of action by which auxin modulates the development of the three seed structures—embryo, endosperm, and seed coat—has recently started to be clarified [15,16,17,18]. In fact, seed formation is driven by auxin that has been synthesized after fertilization. Moreover, the auxin generated in the endosperm is reported to be closely related to seed-coat development. In Arabidopsis, the autonomous development of the seed coat can be activated by either exogenous auxin or ectopic production of auxin in the central cells of unfertilized ovules [19]. Furthermore, the suppression of auxin biosynthesis in the endosperm impaired seed-coat development [19]. More studies have shown that auxin may be transported from the endosperm to the envelope under the regulation of MADS-box transcription factor AGL62, thereby eliminating the inhibitory effect on Polycomb Group (PcG) proteins during seed-coat development [15,19,20]. In addition, auxin application on unpollinated tomato pistils induced defective seed [21], which indicates that the phytohormone auxin plays a very important and conserved role in the regulation of seed-coat formation in different species.

To date, various genes involved in the development of ovule integuments and seed coat have been discovered and identified in *Arabidopsis*. For instance, the *APETALA2 (AP2)* gene, a member of the family of transcription factors encoding AP2/ERF (APETALA2/ethylene-responsive element-binding factors), regulates the morphogenesis of floral meristems and floral organs [22,23,24]. The AP2/ERF transcription factors contain the AP2 DNA-binding domain, which is found in various proteins as a necessary and sufficient condition for binding the GCC box [24]. In addition to floral functions, *AP2* involves the regulation of seed development in *Arabidopsis*, such as seed coat, seed size, seed mass, seed oil, and protein content [25,26]. *ap2* mutants have increased seed size and seed weight, along with a lack of arrest and mucilage in seed coat development and larger outer integument cells than wild-type plants, along with an irregular shape [25,26]. *AUXIN RESPONSE FACTOR 2* (*ARF2*) in Arabidopsis encodes a B3-type transcription factor of the ARF family, which is thought to act as a transcriptional repressor that negatively regulates cell proliferation and expansion [27,28]. The *megaintegumenta* (*mnt*) mutant is an *arf2* loss-of-function mutant whose seeds are larger compared to wild-type seeds, present more cells in the seed coat, and are observed to present additional cell layers in the integuments prior to fertilization [28]. The MADS-box *VvAGL11* gene in grapes, which especially accumulates in the inner seed coat, also modulates seed size and lignification [29,30]. The natural loss-of-function allele of *VvAGL11* prevents proper seed coat differentiation, ultimately leading to endosperm degeneration and cessation of embryo development [31]. Furthermore, another gene, *VvTTG2* (*VvWRKY26*), is reported to regulate seed coat color by modulating vacuolar transport and the accumulation of proanthocyanidin (PA) [32].

Previously, our lab has identified that the ovules of emasculated grapes treated with the plant growth regulator 4-CPA can continue to grow and differentiate to form seeds [14]. This indicates that 4-CPA may play a critical role in the process of ovule development into seeds. In this work, we continue to investigate the effect of exogenous 4-CPA treatment on the grape ovary after emasculation, fruit set, and ovule development and the function of seed-coat-related genes *VvARF2* and *VvAP2*.

## 2. Materials and Methods

### 2.1. Plant Material

The hybrid grape cultivar “Fenghou” (*V. vinifera* × *V. labrusca*) was planted in a nursery at Wenquan Town, Beijing, the People’s Republic of China. All the treatments were conducted over three consecutive years and started with 15-year-old grapevines.

### 2.2. Pharmacological Treatments

The synthetic pesticide 4-chlorophenoxyacetic acid (4-CPA, Sigma-Aldrich, St. Louis, MS, USA), which can modulate grape growth, was employed to induce defective seed generation in this study, with a concentration of 0.08 mM [14]; 4-CPA was dissolved in a solution of 94.9% water, 5% ethanol, and 0.1% Tween 80. Based on different treatments, these inflorescences were mainly divided into the emasculated group (30 inflorescences) and the nonemasculated group (10 inflorescences). In the emasculated group, 20 inflorescences were sprayed with 0.08 mM 4-CPA, and the remaining 10 inflorescences were treated with solvent alone. The 10 inflorescences of the nonemasculated group were used as the controls.

The inflorescence samples were respectively collected 4, 8, 12, 16, and 21 days after treatment (DAT) and divided into 3 groups (each group contained the same number of inflorescences): Group I was fixed with FAA (formaldehyde–acetic acid–ethanol fixative; 50% ethanol, 5% glacial acetic acid, and 5% formaldehyde), and used to make paraffin sections for microscopic observation; Group II was immediately frozen in liquid nitrogen after collection and stored at −80 °C for RNA extraction; Group III was prepared for in situ hybridization and fixed with PBS that contained 4% (*w*/*v*) formaldehyde at 4 °C.

### 2.3. Organizational Structure Observation and Statistical Analysis

The ovaries or young fruits of Group I, which have been fixed by FAA, were firstly dehydrated via passing through an ethanol series and then embedded in paraffin. These samples were cut into 8-μm sections and stained with Safranin and Fast green. An Olympus CX31 microscope was used to observe and photograph the sample’s organizational structure.

ImageJ software was used to measure the ovule integument cell diameter and thickness in the ovary of young fruit at 4, 8, 12, and 16 DAT.

### 2.4. RNA Extraction and Quantitative Real-Time PCR Analysis

The total RNA was extracted from “Fenghou” grape ovaries/fruits of Group II using a modified CTAB method, and they were purified with RNase-free DNase I (Takara, Kyoto, Japan). An atomic ultraviolet spectrophotometer was used to determine RNA concentration. First-strand cDNA synthesis was initiated from 2 µg total RNA using reverse transcriptase (Promega, Madison, WI, USA) and oligo dT primers according to the manufacturer’s instructions. We performed quantitative real-time PCR (qRT-PCR) with the cDNAs on an ABIPRISM 7500 based on the manufacturer’s instructions. The reaction conditions were described previously [14]. All experiments were performed in three replicates and two technical replicates. We used the Primer software 5.0 (PREMIER Biosoft International, Palo Alto, CA, USA) to design primers for *VvAGL11*, *VvTTG2*, *VvAP2*, and *VvARF2*. The relative transcription levels were calculated using the 2^−ΔΔCt^ method [33] and normalized to the relative transcript levels of the housekeeping gene *VvUBQ* as an internal standard. All quantitative primers are listed in Table S1.

### 2.5. In Situ Hybridization

Probes for *VvAP2* and *VvARF2* were PCR-amplified from cDNA templates with gene-specific primers. We sequenced the PCR product and found it was composed of 500 bp of *VvAP2* and 500 bp of *VvARF2*. The DNA fragment showed 99% similarity to the published sequence. The PCR product was ligated into the pMD-18 vector (Takara Bio, Beijing, China). According to the manufacturer’s instructions, antisense probes were synthesized using the DIG RNA labeling kit (Sp6/T7; Roche, Basel, Switzerland). As described by Drews et al. (1991), digoxigenin labeling was used for RNA probe labeling, tissue preparation, and in situ hybridization [34].

### 2.6. Statistical Analysis

Kruskal–Wallis tests and Student’s *t*-tests were conducted to assess statistical significance among different treatments. Significant differences between the means were based on the least significant method when the F-values were significant (*p* < 0.05).

## 3. Results

### 3.1. 4-CPA Inhibits Fruit Growth But Not Fruit Setting

**Figure 1:**
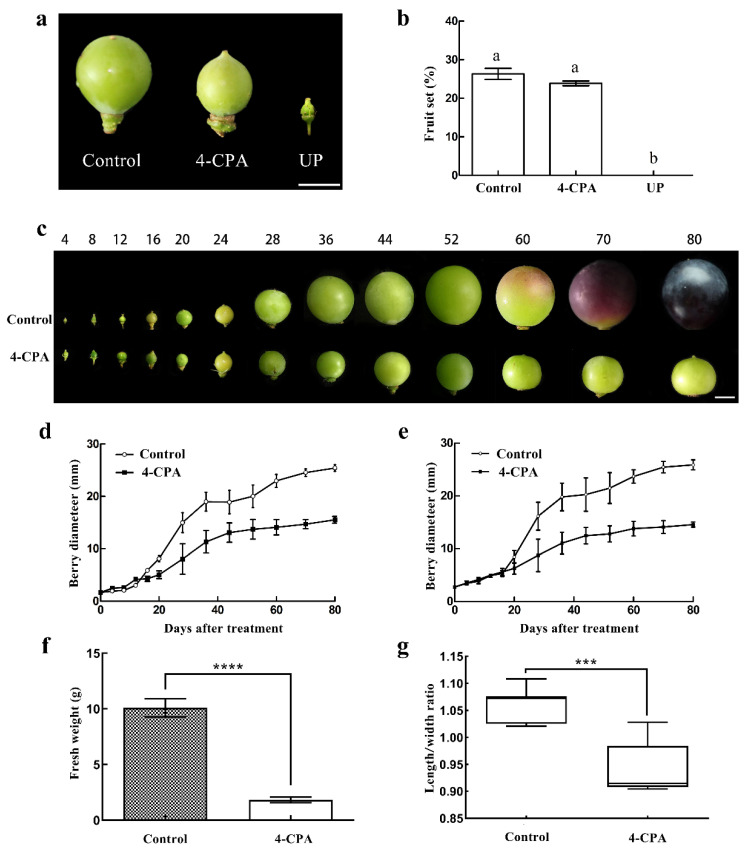
Effect of 4-CPA (4-chlorophenoxyacetic acid) treatment on fruit setting and fruit development of “Fenghou” grapes. (**a**) Control, 4-CPA treatment, and nonpollinated ovary at 28 DAT; (**b**) Control, 4-CPA treatment, and nonpollinated fruit setting; (**c**) Control and 4-CPA treatment of fruit development in different periods; (**d**,**e**) Control and 4-CPA treatment of the changes in the horizontal and vertical diameters of the fruits at different developmental stages; (**f**) the average fruit weight of Control and 4-CPA treatment at 80 DAT; (**g**) Control and 4-CPA treatment fruit aspect ratio at 80 DAT. The scale bar of (**a**,**c**) = 1 cm. Abbreviations: 4-CPA, 4-CPA treatment; UP, nonpollination treatment. Significant differences (*p* < 0.05) between treatments are indicated by different letters according to the Kruskal–Wallis test. Significance analysis was conducted with two-tailed Student’s *t*-tests (*** *p* < 0.001; **** *p* < 0.0001).

To explore the regulatory role of 4-CPA on “Fenghou” grape fruit setting and fruit growth, we used exogenous 4-PCA sprayed on the grape ovary at 8 DBA. At 28 DAT, the ovary diameter of 4-CPA-treated grapevines and control grapevines were 8 and 15 mm, respectively. When grapevines were emasculated but not sprayed with any 4-PCA, the development of the ovaries ceased (Figure 1a). A similar fruit setting rate was detected in 4-CPA-treated grapevines and control grapevines: 23.87% and 26.33%, respectively. In agreement with our observations on ovary development, the untreated fruit setting rate after emasculation was 0 (Figure 1b). On the side of fruit growth and development, the control fruits started to be colored at 60 DAT and were fully colored and mature at 80 DAT. Compared with control fruits, the 4-CPA-treated fruits were smaller after 20 DAT, and the fruit shape was oblate; they were not colored normally until 80 DAT (Figure 1c). The fruit growth curve also revealed that the horizontal and vertical diameters of the control fruits were both larger than 4-CPA-treated fruits from 20 DAT. Before 16 DAT, the horizontal diameter of the 4-CPA-treated fruit was larger than that of the control fruit, but the vertical diameter of the fruit was not significantly different between both (Figure 1d,e). Furthermore, the development of 4-CPA-treated fruits was almost stagnated after 44 DAT. Additionally, there were no significant differences in fruit size at 80 DAT. The average single-fruit weight of 4-CPA-treated fruits was 1.83 g, which was much lower than the control fruit (10.1g) (Figure 1f). The fruit shape index after the 4-CPA treatment was lower than that of the control fruit (Figure 1g). Thus, our results revealed that the 4-CPA was able to reduce fruit growth after 20 DAT but had no impact on fruit set.

### 3.2. The Effect of 4-CPA Treatment on Seed Growth and Development 

**Figure 2:**
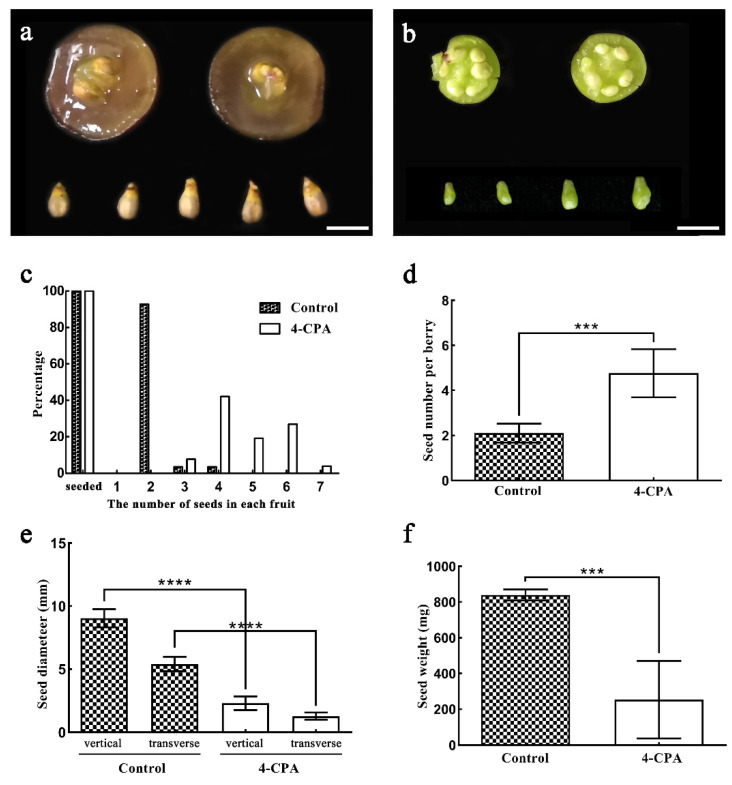
Effect of 4-CPA treatment on the growth and development of grape seeds. (**a**) Fruit and seed form of the control; (**b**) fruit and seed morphology after 4-CPA treatment; (**c**) percentage of seeds in each fruit and the proportion of fruits with different numbers of seeds; (**d**) average number of seeds; (**e**) vertical and transverse diameter of the seeds; (**f**) average seed weight. The scale in (**a**) and (**b**) is 1 cm. Significance analysis was conducted with two-tailed Student’s *t*-tests (*** *p* < 0.001; **** *p* < 0.0001).

In addition to inhibiting fruit growth, surprisingly, seed-like products were observed in 4-CPA-treated fruits. These products looked similar to normal seeds, albeit being smaller and in larger quantities (Figure 2a,b). For instance, 92.86% of control fruit only contained 2 seeds, 3.57% had 3 or 4 seeds, and no fruit contained more than 4 seeds. In contrast, more seed-like products were found in 4-PCA-treated fruits: the respective percentage of 2, 3, 4, 5, 6, and 7 seed-like products was 0, 7.69%, 42.31%, 19.23%, 26.92%, and 3.85%, respectively (Figure 2c). The average number of seeds or seed-like products per fruit for the control and 4-CPA treatment was 2.1 and 4.8, respectively (Figure 2d). In terms of the size of seeds or seed-like structures, the transverse diameters of the seeds or seed-like products obtained from the control and 4-CPA treatment were 5.4 and 1.3 mm, and the vertical diameters of the seeds were 9.0 and 2.3 mm, respectively (Figure 2e); the average single-seed weight of the seeds was 839.6 and 253.8 mg, respectively (Figure 2f). These results indicate that the average number of seed-like products per fruit after 4-CPA treatment is more than that of control fruit and that all fruit after 4-CPA treatment contained three or more seed-like products, whereas the majority of the control fruit contained two seeds. However, the seed-like products obtained after 4-CPA treatment were smaller than those obtained from the control.

**Figure 3:**
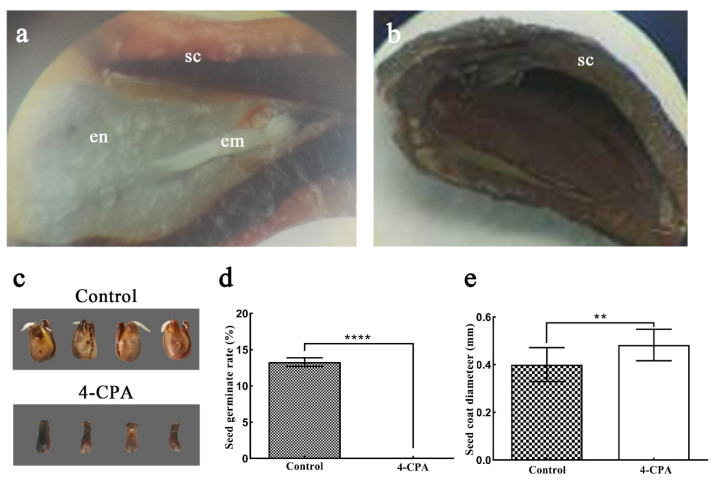
The effect of 4-CPA on the germination of grape seeds. (**a**) Freehand section of a control seed, showing a developed endosperm and embryo structure; (**b**) a seed treated with 4-CPA, without the embryo and endosperm structure; (**c**) seed germination; (**d**) germination rate statistics; (**e**) seed coat thickness. Abbreviations: sc, seed coat; en, endosperm; em, embryo. Significance analysis was conducted with two-tailed Student’s *t*-tests (** *p* < 0.01; **** *p* < 0.0001).

To detect whether there was apomixis under 4-PCA treatment, anatomical observations and germination tests were conducted on these seed-like products. There were no embryo and endosperm in the seed-like products obtained after 4-CPA treatment; only a thicker seed coat was detected (Figure 3a,b,e). Furthermore, these products could not germinate at all. In comparison, the germination rate of normal seeds was 13.3% (Figure 3c,d). Thus, we named the seed-like products induced by 4-PCA “defective seeds”, which were not the result of apomixes.

### 3.3. Observation of Histological Structure of Defective Seed

Since the auxin produced in the endosperm can drive seed coat development in Arabidopsis [19], we assumed that the formation of defective seeds (mainly containing seed coats) was the result of 4-PCA mimicking endosperm auxin. Thus, we further counted the number of cell layers and measured the cell size of the integument in the ovules after 4, 8, 12, and 16 DAT (Table 1). There was almost no difference in the number of cell layers in the inner integument between the 4-CPA treatment and the control. Additionally, the cell diameter and cell area of 4-CPA-treated seeds were slightly larger than that of control. However, the number of cell layers, cell diameter, and cell area in the outer integument were significantly increased under the 4-CPA treatment.

**Figure 4:**
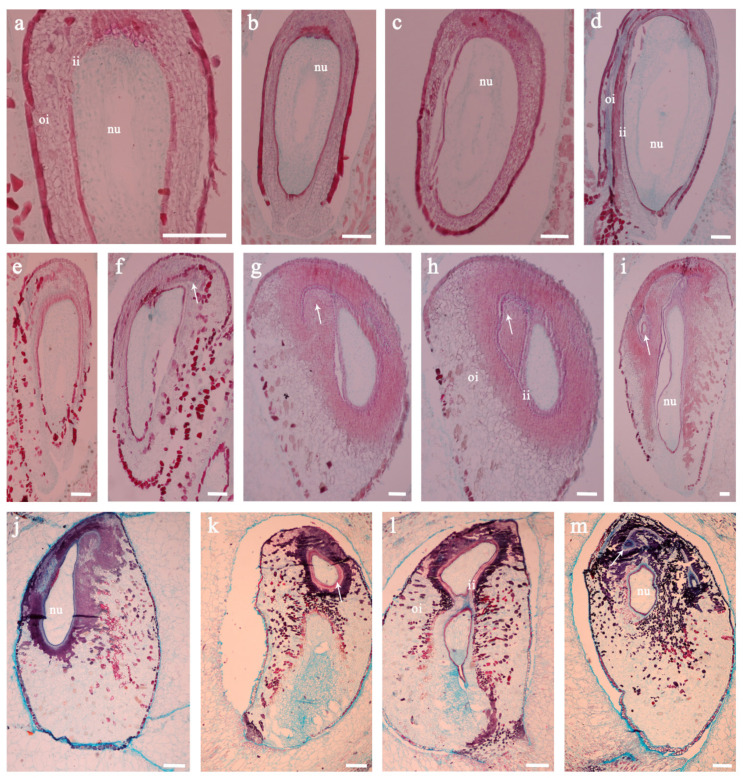
The effect of 4-CPA treatment on ovule development at 4 to 21 DAT. (**a**–**d**) are longitudinal sections of ovules at 4, 8, 12, and 16 DAT, respectively; (**e**–**g**), (**h**), (**i**), (**j**) and (**k**–**m**) are longitudinal sections of ovules at 4, 8, 12, 16, and 21 DAT, respectively. The white arrow represents the new ovule-like structure. Abbreviations: nu, nucellus; oi, outer integument; ii, inner integument. The scale in the figure represents = 200 μm.

Observation of the structure of the ovules at different stages of development revealed that the integument had 8–9 layers of cells in the ovary before flowering. At this time, the cells were arranged neatly, the nucellus also had about 8 layers of parenchyma cells, and the embryo sac began to develop (Figure 4a). The embryo development started at the end of pollination and fertilization. During this process, the number of integument cells in the pollinated and fertilized ovary did not change much, but the nucellus tissue continued to grow (Figure 4b–d). At 4 DAT (before flowering), the number of integument cells treated with 4-CPA was not much different from the control (Figure 4e). However, cell redifferentiation began to appear on the top of the ovule at 8 DAT (Figure 4f), and re-differentiated new ovule primordia appeared at 12 DAT (Figure 4g), resulting in abnormally thickened outer cells of the ovule (Figure 4h). Furthermore, some ovule primordia, induced by differentiation at the apex of the ovule, developed into a young ovule-shaped structure (Figure 4i). At 16 DAT, the integument tissue after 4-CPA treatment was abnormally developed, but the nucellus tissue was significantly atrophied (Figure 4j). Until 21 DAT, the developing new ovule-like structure could also be observed in the developed integument tissue after 4-CPA treatment (Figure 4k). Meanwhile, the integument cells were being differentiated at the top of the ovule (Figure 4l), which protruded into a seed shape (Figure 4m). These findings indicate that 4-CPA can stimulate the division and continuous development of integument cells and promote the redifferentiation of cells at the top of the ovule to form a structure similar to a new ovule.

### 3.4. Auxin Regulates the Expression and Location of Grape-Ovule-Related Genes

**Figure 5:**
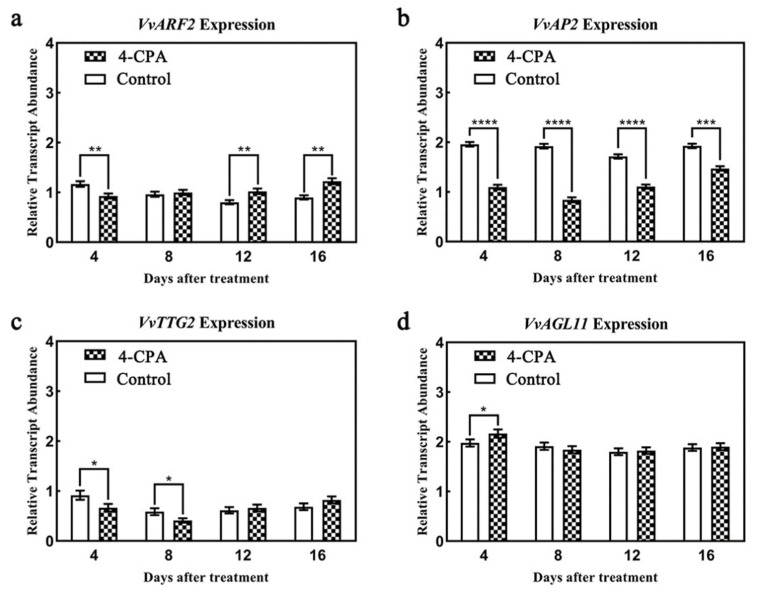
The expression changes of *VvARF2*, *VvAP2*, *VvTTG2*, and *VvAGL11* genes after 4-CPA treatment. (**a**) is the expression change of *VvARF2* gene after 4-CPA treatment; (**b**) is the expression change of *VvAP2* gene after 4-CPA treatment; (**c**) is the expression change of *VvTTG2* gene after 4-CPA treatment; (**d**) is the expression change of *VvAGL11* gene after 4-CPA treatment; Significance analysis was conducted with two-tailed Student’s *t*-tests (* *p* < 0.05; ** *p* < 0.01;*** *p* < 0.001; **** *p* < 0.0001).

Our results above indicate that 4-CPA induces cell division and redifferentiation of the integument, resulting in abnormal thickening and cell enlargement. Meanwhile, it also induces the integument to form a seed coat. To investigate the related gene expression changes during this process, we firstly used protein sequences of genes known to be associated with integument cell division and development in Arabidopsis as queries for BLAST searches of the grapevine genome database (http://plants.ensembl.org/Vitis_vinifera/Info/Index) (accessed on 25 June 2020). Genes *VvARF2*, *VvAP2*, *VvTTG2*, and *VvAGL11* were retrieved from the grapevine genome, respectively. Thus, the transcriptional level changes of integument-development-associated genes after 4-CPA treatment were detected by qRT-PCR. Compared with the control, we found the expression of *VvARF2* was lower at 4 DAT but higher for 8–16 DAT (Figure 5a). The *VvAP2* expression was significantly lower than the control at all tested time points after 4-CPA treatment (Figure 5b). Consistently, *VvTTG2* induction levels were also lower than the control at 4 and 8 DAT (Figure 5c). However, *VvAGL11* transcription levels did not show any differences between the 4-CPA treatment and the control (Figure 5d).

**Figure 6:**
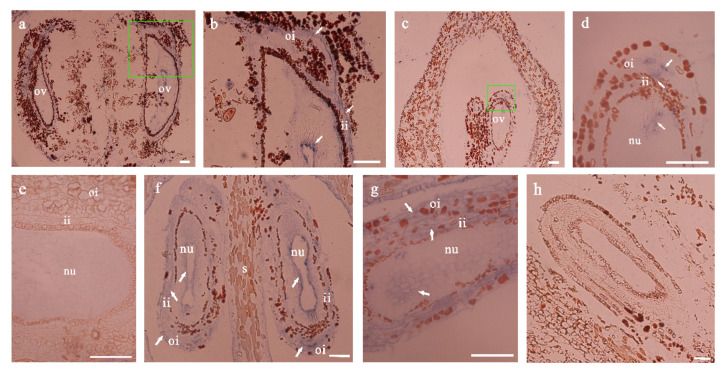
In situ hybridization analysis of *VvARF2* and *VvAP2* genes in ovules after 4-CPA treatment. The location of *VvARF2* mRNA in the ovules after 12 days of 4-CPA treatment (**a**) and control (**c**); their respective zoomed pictures are given in (**b**) and (**d**). The location of *VvAP2* mRNA in the ovules of 4-CPA treatment after 12 days (**e**) and control (**f**–**g**). (**h**) is the negative control. White arrows indicate a hybridization signal. Abbreviations: ov, ovule; nu, nucellus; s, septum; oi, outer integument; ii, inner integument. Scale bar in the figure = 100 μm.

Since the integument redifferentiation induced by 4-CPA treatment mainly occurred at 12 DAT, the expression of genes *VvARF2* and *VvAP2* also showed a significant difference during this time; we assumed that these two genes might be involved in a signaling pathway activated by 4-CPA. Therefore, we used in situ hybridization to locate the expression positions of *VvARF2* and *VvAP2*. A strong *VvARF2* mRNA hybridization signal was detected in the nucellus and inner and outer integument tissues 12 days after 4-CPA treatment (Figure 6a,b), while the hybridization signal was much weaker in the same positions in the control ovules (Figure 6c,d). In 4-CPA-treated ovules after 12 days, almost no *VvAP2* hybridization signals were detected (Figure 6e,f). However, the *VvAP2* hybridization signal was detected in the control nucellus and integument tissue (Figure 6g). Thus, the qRT-PCR results were in line with in situ hybridization, namely, that 4-CPA treatment increased the expression of the *VvARF2* gene and inhibited the expression of the *VvAP2* gene in the ovule, which affected the number of integument cell layers and cell size, which, in turn, affected seed coat development.

## 4. Discussion

Our study has shown that the 4-CPA-induced defective seeds in parthenocarpic grapes are caused by promoting integument development and seed coat formation. This conclusion was generated by the observation that defective seeds were present in fruit formed from unpollinated ovaries under 4-CPA treatment and that such defective seeds contained no embryo and endosperm, only the seed coat (Figure 2 and Figure 3). The development of the seed coat is triggered by auxin that has been synthesized in the developing endosperm and exported to the integument after fertilization [15,19]. Further, the results of the present study support the idea that the plant hormone auxin is a key factor in triggering seed coat formation in grapes.

The seed coat of plants originates from the integument tissue of the ovule [35]. Generally, the integument tissue is divided into outer integument and inner integument, which enclose the nucellus and female gametophyte. After fertilization, the integument tissue grows cooperatively with the embryo and endosperm [36]. Finally, the seed coat is formed to build a barrier to protect the inner seeds [37]. The thickness of the seed coat, which is determined by the number of integument cell layers, can affect seed germination, dormancy, and death [38,39]. Since we have found the 4-CPA can stimulate the division of grape integument cells, this might explain the occurrence of the thicken seed coat under 4-CPA application. Furthermore, in *Arabidopsis*, integument formation and development are largely regulated by the plant hormone auxin through the modulation of proximal–distal axis polarity [40,41]. Consistently, 4-CPA promotes the redifferentiation of apical cells of the ovule to form new ovules, which might be the regulatory effect of polarity, leading to the formation of more seed-like products. In a word, our findings of 4-PCA-regulated seed development in grapes are largely overlapped with auxin’s regulation effects on *Arabidopsis*. However, the changes of thickness of seed coat and seed numbers under auxin regulation are clear and measurable in grapes.

Furthermore, tissue observation is discussed together with the transcriptional changes of several seed-coat-associated genes in this study. The qRT-PCR results revealed that *VvARF2* gene expression was upregulated while *VvAP2* gene expression was downregulated (Figure 5a,b), indicating that the two genes play distinct roles under 4-CPA treatment. VvARF2 and VvAP2 are homologs of *Arabidopsis* ARF2 and AP2, respectively [42,43]. An increment of the number of integument cell layers and thickened seed coats were detected in *Arabidopsis ARF2* mutant [28]. In contrast, when 4-CPA induced an increase in the number and size of integument cell layers, the expression of the *VvARF2* gene increased in the grapes. Additionally, its hybridization signal was present on both the integuments and at the nucellus. We hypothesized that the opposite *VvARF2* expression in grapes might be caused by the strong accumulation of *VvARF2* mRNA signals at the nucellus. The Arabidopsis *ap2* mutant had enlarged outer integument cells [25,26,44]. In agreement with this, we found that the outer integument cells of defective seeds were enlarged. The decreased *VvAP2* expression was in line with the finding that its mRNA hybridization signal was diminished on the integument. Together, these findings suggest that *AP2* is functionally conserved in woody plants.

**Figure 7:**
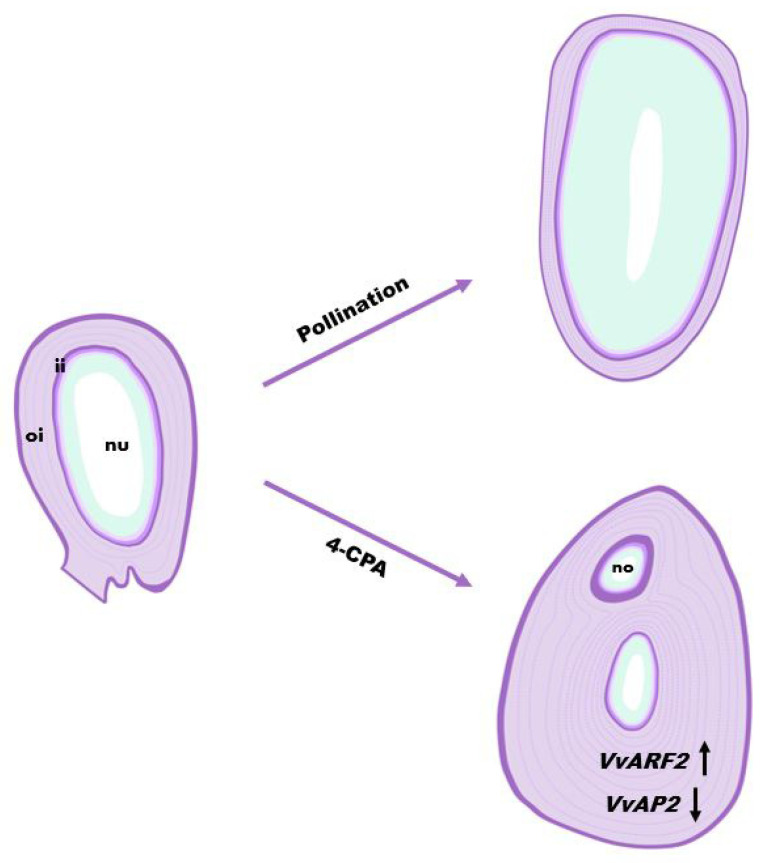
Scheme of 4-CPA inducing the formation of defective grape seeds. 4-CPA treatment upregulated *VvARF2* gene expression and inhibited *VvAP2* gene expression, accelerated cell division and cell redifferentiation of the integument tissue, increased the number of integument cell layers in the ovule, and formed new ovules. Abbreviations: no, new ovules; nu, nucellus; oi, outer integument; ii, inner integument.

In this study, we demonstrate that 4-CPA-induced defective seeds were caused by regulating the division and continuous redifferentiation of integument cells in “Fenghou” grapes. Our findings reveal that the 4-CPA treatment could upregulate *VvARF2* gene expression, inhibit *VvAP2* gene expression, and accelerate cell division and cell differentiation in the integument tissues. These events induced the increment of integument cell layers in the ovules and maintained continuous division and growth, resulting in the formation of defective seeds with only thick seed coats. Meanwhile, the redifferentiation of the integument tissue allowed the formation of new ovules, leading to a larger number of seeds in the ovary after 4-CPA treatment (Figure 7). Overall, the study elucidated specific functions and mechanisms of 4-CPA on seed development in grapes.

## Figures and Tables

**Table 1:** Changes in the number of cell layers, cell diameter, and cell size of the inner and outer integuments after 4-CPA treatment.

		Inner Integument			Outer Integument		
		Cell Layers	Cell Diameter (μm)	Cell Area (μm^2^)	Cell Layers	Cell Diameter (μm)	Cell Area (μm^2^)
4DAT	Control	2.67 ± 0.47	4.42 ± 0.45	61.93 ± 13.11	5.44 ± 0.50	7.18 ± 0.29	162.29 ± 13.06
	4-CPA	2.78 ± 0.42	4.75 ± 0.40	71.44 ± 12.03	7.44 ± 0.50	11.74 ± 1.06	436.26 ± 80.40
8DAT	Control	2.67 ± 0.47	4.51 ± 0.36	64.30 ± 10.01	5.78 ± 0.63	7.44 ± 0.52	174.55 ± 24.53
	4-CPA	3.00 ± 0.00	5.44 ± 0.41	93.56 ± 13.12	8.11 ± 0.31	12.29 ± 1.02	477.76 ± 75.40
12DAT	Control	2.89 ± 0.31	3.39 ± 0.30	36.32 ± 6.60	6.33 ± 0.47	9.32 ± 0.57	273.83 ± 32.73
	4-CPA	3.00 ± 0.00	5.10 ± 0.19	81.89 ± 6.19	12.22 ± 0.92	15.04 ± 0.49	711.56 ± 45.95
16DAT	Control	3.00 ± 0.00	3.44 ± 0.25	37.27 ± 5.27	6.44 ± 0.68	11.16 ± 0.42	391.87 ± 29.35
	4-CPA	3.00 ± 0.00	4.88 ± 0.35	75.32 ± 10.81	13.00 ± 0.94	15.07 ± 1.28	718.75 ± 119.22

## Supplementary Materials

The following are available online at https://www.mdpi.com/article/10.3390/biom11040515/s1, Table S1: Primers used in this study.
