# Supplementary material for: 4-CPA (4-Chlorophenoxyacetic Acid) Induces the Formation and Development of Defective “Fenghou” (Vitis vinifera × V. labrusca) Grape Seeds
Source: Biomolecules. 2021 Mar 30;11(4):515. doi: 10.3390/biom11040515 (PMC8067128; doi:10.3390/biom11040515)
Supplement: Supplementary file 1 [file biomolecules-11-00515-s001.pdf]

biomolecules

Table S1. Primers used in this study.

Name	Sequence(5 ‘→3 ‘)	Purpose
VvUBQ-F	GCTCGCTGTTTTGCAGTTCTAC	RealTime-PCR Specific houskeeping
VvUBQ-R	AACATAGGTGAGGCCGCACTT	
VvARF2-F	AAACCTAGGACAAGCCCTGC	RealTime-PCR Specific *VvARF2*
VvARF2-R	AATCCCAACTATGGTGCCCG	
VvAP2-F	TTCAGACCTAAGTTCAACCTGC	RealTime-PCR Specific *VvAP2*
VvAP2-R	TTTGGTGAGCTGAGCTGTGG	
VvTTG2-F	CAGAGACAGTTAGTTGGCCGT	RealTime-PCR Specific Vv*TTG2*
VvTTG2-R	AATGGAGGCTTGGGGATGTG	
VvAGL11-F	AACAAACGAATCTCAAGCAATCAAGC	RealTime-PCR Specific Vv*AGL11*
VvAGL11-R	TGATCTCGATCTTTCCTCTCCCC	
VvARF2-F	CGGAAGGGCAGAAAGGTCAT	[1]ISH probe PCR
VvARF2-R	TTGGCAACCAACTCCTGTGT	
VvAP2-F	GTTGCCACCGCCAAGTCA	ISH probe PCR
VvAP2-R	CTTCCCATCTTCCGCACT	
